# Quadriceps handheld dynamometry during the post-ICU trajectory: using strictly the same body position is mandatory for repeated measures

**DOI:** 10.1186/s40635-023-00523-5

**Published:** 2023-07-03

**Authors:** Anne-Françoise Rousseau, Nadia Dardenne, Isabelle Kellens, Stephen Bornheim, Benoit Misset, Jean-Louis Croisier

**Affiliations:** 1 Intensive Care Department and Burn Center, University Hospital, University of Liège, Sart-Tilman B35, Hippocrate Avenue 1, 4000 Liège, Belgium; 2 Biostatistics Center (B-STAT), University Hospital and University of Liège, Liège, Belgium; 3 Department of Sport Sciences and Rehabilitation, University of Liège, Liège, Belgium

**Keywords:** Quadriceps strength, Dynamometry, Reliability, Equation, Position, Conversion

## Abstract

**Background:**

The level of quadriceps strength (QS) generated in the supine or seated position is not similar. For QS follow-up from intensive care unit (ICU) stay to recovery, getting comparable measures is essential. This study aimed to develop and validate new equations for estimating QS in a given position based on the measurement taken in another one.

**Methods and results:**

Isometric QS was measured using a handheld dynamometer and a standardized protocol in a supine and in a seated position. In a first cohort of 77 healthy adults, two QS conversion equations were developed using a multivariate model integrating independent parameters such as age, sex, body mass index (BMI) and baseline QS. These equations were tested in two cohorts for external validation, using the interclass correlation coefficient (ICC) and Bland–Altman graphical method. Only one was validated in the second cohort (62 different healthy adults): the ICC was 0.87 (95% CI 0.59–0.94) and the bias was − 0.49 N/Kg (limits of agreement: − 1.76–0.78 N/kg). However, this equation did not perform well in the third cohort (50 ICU survivors): the ICC was 0.60 (95% CI 0.24–0.78), and the bias was − 0.53 N/Kg (limits of agreement: − 1.01–2.07 N/kg).

**Conclusions:**

As no conversion equation has been validated in the present study, repeated QS measurements should be performed strictly in the same standardized and documented position.

## Introduction

There is evidence that physical impairment, partly due to muscle weakness, is an important problem in critically ill patients who survive a stay in an intensive care unit (ICU) [1]. However, evidence on how and when to intervene is lacking, probably linked with the diversity of methods that can be used to document the physical impairment [2]. Quantitative assessment of muscle status may be useful for the diagnosis and follow-up of ICU acquired weakness [3]. The quadriceps muscle group is essential for standing, sitting, and walking, so its strength has been related to limb function [4–6]. Considering quadriceps strength as a relevant physical outcome thus makes sense. Isokinetic dynamometry is considered as the gold standard for muscle strength measurement. Unfortunately, portability, procedure and costs are barriers for its use at ICU bedside. In the critical care setting, isometric testing using a handheld dynamometer is a portable, light, and inexpensive option, with good sensitivity to quantify strength changes over time [7]. Using the same method from ICU stay to recovery makes sense from a follow-up point of view, aiming to get comparable strengths.

A highly standardised protocol of quadriceps strength (QS) measurement in a modified supine position with the tested leg in 45° hip flexion and 40° knee flexion has been previously validated [8, 9]. It can be advantageously performed as soon as the patient is cooperative, in the ICU setting. However, due to the required equipment, it is more difficult to use this protocol outside the ICU, such as in a post-ICU follow-up consultation. After ICU discharge, the most suitable position for quadriceps testing is the seated position with the tested leg at 90° hip flexion and 90° knee flexion. In the available literature, quadriceps dynamometry is mostly performed in the seated condition [10, 11].

For a longitudinal follow-up of muscle strength, getting consistent and comparable measures is essential, both for clinical and research purposes. In a recent experimental approach of QS measurement in healthy volunteers, it has been demonstrated that body position influenced the level of generated QS: quadriceps was less efficient in the seated position, compared to the supine position [12]. In other words, strength measured in one position cannot be compared to the strength measured in another position. These findings have a concrete impact in case of repeated measurements: the testing position should be the same for all measurements.

In this prospective study, we tried to overcome this limitation and to allow QS comparisons. The first aim of the present study was to develop new equations for estimating QS in a given position based on the measurement in another one. The second aim was to test the generated equations in two other cohorts of either healthy volunteers or ICU survivors.

## Methods

This study was conducted in 2021 and 2022, after approval by the local Ethics Committee of our University Hospital (National Ref B7072021000006, Local Ref 2021/45, 31st March 2021). The participants were fully informed of the study’s purpose, procedures, and limited risks. Informed consent was obtained prior to enrolment.

The study was composed of three parts: the first part was dedicated to the development of the equations and internal validation in healthy volunteers, the second part was dedicated to external validation in healthy volunteers, and the third part was dedicated to external validation in ICU survivors.

### Participants

For the first and second parts of the study, two distinct convenience samples of healthy volunteers were recruited among the medical and paramedical ICU staff members, and among subjects who attended upper limb physiotherapy sessions in our hospital (i.e.: development cohort and validation cohort of volunteers). The same patient could not be included twice in this study. Inclusion criteria was age ≥ 18 years. Exclusion criteria included total hip or knee arthroplasty in the dominant limb, pre-existing myopathy or polyneuropathy, and a history of traumatic spine or lower limb injury within the past 6 months.

For the third part of the study, a convenience sample of ICU survivors were recruited among adults who were discharged from our ICU after a stay of at least 48 h. Exclusion criteria were RASS (Richmond Agitation and Sedation Scale) score > 1 or < − 1, coma, total hip or knee arthroplasty in the dominant limb, unauthorized weight-bearing on the dominant leg, an open wound at the lower anterior face of the dominant leg, pre-existing myopathy or polyneuropathy, para- or tetraparesis, para- or tetraplegia, or refusal to participate.

### Quadriceps strength testing

**Fig. 1:**
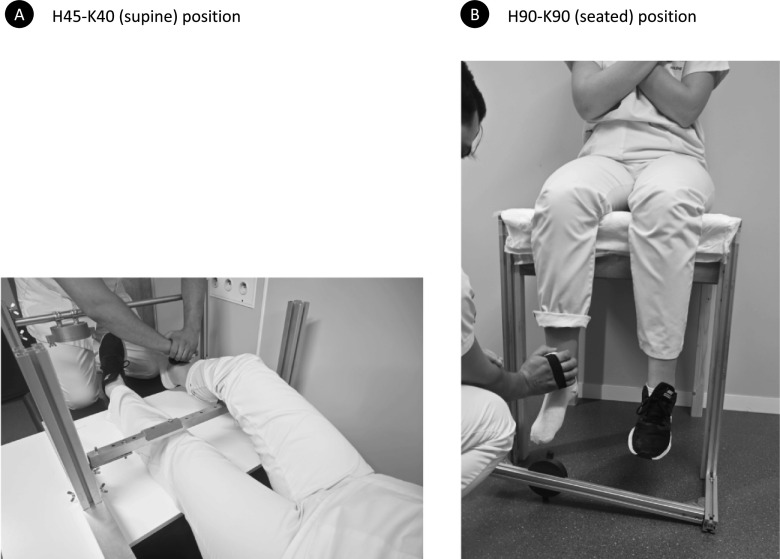
Illustration of the testing structure, participant positioning and operator positioning in the H45–K40 (**A**) and H90–K90 (**B**) positions

Maximal isometric voluntary quadriceps contraction was assessed using a handheld dynamometer (MicroFet2^®^, Hoggan Health Industries, West Jordan, UT, USA) with a curved transducer pad. The same device was used for all tests. In each cohort, the same trained examiners (physiotherapists) performed all strength measurements. The highly standardised protocol is detailed in a previously published validation study [8]. Intra-observer reliability has been demonstrated in that princeps study including patients with critical illness [8]. A high inter-observer reliability has also been demonstrated for other devices in critically ill patients [10] and for the same MicroFet2 in a healthy elderly population [13]. The dominant limb was tested, defined as the reported kicking leg [14]. The operator was positioned in the front of the patient and held the MicroFET2 in his/her hands, withstanding the subject’s movement (knee extension). The MicroFET2 was localized front of the ankle, two centimetres above the external malleolus level (Fig. 1). The protocol consisted of three consecutive maximal contractions, preceded by three progressively intensified warm-up trials. Subjects were first shown the movement to be tested (“push against the dynamometer by attempting to perform a knee extension”) and then asked to perform it to confirm their understanding and finally did the warm-up. The three measurements were then performed with 30 s intervals between contractions. Subjects were asked to gradually increase the intensity of their contraction up to their maximum effort that had to be sustained for 6 s. The operator provided standardised encouragements (“Ready? Push! Build it up! Push harder! Harder! Harder! Harder! Stop! – with one order per second) to ensure maximal effort during each trial. As the protocol aimed to give the participants the opportunity to reach their maximal strength and not to test their capacity of repeating a muscular effort, the best performance out of the 3 measurements was considered for the analysis. Muscle strength was expressed in Newton (N). In order to reduce inter-individual variability and minimize the effect of subject weight on muscle strength, absolute strength was normalized according to actual body weight (expressed in N/kg).

### Patient position

Measurements were performed in two positions (supine and seated positions) with the subjects’ arms crossed on their thorax. In the supine position (Fig. 1A), limb position was standardized using an adjustable system of vertical and horizontal bars, aimed at getting a 45° hip flexion and a 40° knee flexion (H45–K40) and to maintain the mandatory position throughout the procedure. For comfort purposes, the back of the knee of the tested limb rested on a solid cushion, that could not be flattened. In the sitting position (Fig. 1B), thighs rested on a wooden stand without a backrest for volunteers, or over the edge of the bed without a backrest for ICU survivors. Subject’s legs were hanging unsupported, with their hip and knee flexed at 90° (H90–K90). Correct limb position was confirmed using a long-armed goniometer. The order of the tests was randomly assigned. The measurement in each position was conducted with a ten-minute interval in order to prevent muscle fatigue.

### Other descriptive data

Age (years), gender, weight (kg), height (cm), body mass index (BMI, kg/m^2^) were recorded.

### Analysis

The SAS 9.4 and R programs were used for the analysis of the data collected in this study. Qualitative parameters were expressed as counts and percentages. Normality of quantitative parameters was assessed using the Shapiro–Wilk test. As some quantitative parameters were not normally distributed, results were expressed as medians with first and third quartiles [Q1–Q3]. Data were compared using the Mann–Whitney test, Kruskal–Wallis, Fisher’s exact test or Chi-squared test when appropriate. Comparison of QS in the two positions within a cohort was performed using the Wilcoxon signed-rank test.

In the first part of the study, a priori defined variables (age, sex, BMI, and raw QS in H45–K40 position) were tested against measured relative QS in either the H90–K90 or H45–K40 position using a univariate analysis. Factors found to have significant association (*p* < 0.05) were put into a multiple linear regression model for analysis. Independent parameters were used to formulate the new equations. Tertiles of raw QS in H45–K40 position were used to qualify participants strength as lower, middle or upper tertiles of strength, as no thresholds have been defined so far to distinguish weak from strong individuals. Residual error plots and coefficient of determination (R^2^) were considered for assessing the predictive power of equations. The new equations were then tested in the development cohort. Estimated and measured QS were compared using a paired t-test or Wilcoxon signed rank test. In order to assess accuracy of the new equations, Bland–Altman plots were performed for each equation against measured strengths, indicating bias (i.e., the mean percent difference) and agreements between estimated and measured QS. The reliably was assessed with the intraclass coefficient correlation (ICC: two way random effect, absolute agreement, single rater/measurement) and its 95% confidence interval (95% CI). The reliability was considered as acceptable or excellent with an ICC 0.75–0.9 or ≥ 0.9, respectively [15].

In the second and third parts of the study, the equations which best performed were tested on two other validation cohorts (i.e.: volunteers and ICU survivors). The same statistical method was applied to test the new equations.

A *p* value < 0.05 was considered statistically significant.

## Results

### First part of the study: development of the new equations

In the development cohort, 77 participants (34 males, 44.2%) were included. The median age of the subjects was 42 [25–57] years; median height, 170 [164–179] cm; median weight, 75 [64–83] kg; and median body mass index, 24.3 [22.2–27] kg/m^2^. The measured raw and relative QS in the two positions are shown in Table 1. Patients were categorized as weak, moderate or strong according to the tertiles of raw QS in H45–K40: < 362N, 362-483N and > 483N, respectively.

**Table 1:** Raw and relative quadriceps strength in the two cohorts

	Development cohort n = 77	Validation cohort–healthy volunteers n = 62	Validation cohort–ICU survivors n = 50
H45–K40 (N)	428.4 [338.1–508.1]	404.9 [337.5–501.8]	225.4 [172.3–284.4]
H45–K40 (N/kg)	5.91 [4.86–7.38]	5.68 [4.79–6.75]	2.89 [2.25–3.59]
H90–K90 (N)	307.4 [248.7–381.7]	391.7 [334.4–501.2]	242.9 [187.4–302.6]
H90–K90 (N/kg)	4.38 [3.4–5.66]	5.82 [4.57–6.54]	2.83 [2.51–3.74]

Relative QS in H45–K40 was significantly associated with relative QS in H90–K90 (p < 0.001), age (p = 0.001), BMI (p < 0.001) and raw QS in H45–K40 in the moderate and strong tertiles (p = 0.001 and p < 0.001, respectively). Sex was not associated with the dependant variable (p = 0.20).

The first step was simply to determine the ratio between relative QS in H45–K40 position and in H90–K90 position. However, agreement between measured and estimated relative QS using such ratio was unsatisfactory. A second step was to search for a more complex model. The final equation estimating relative QS in H45–K40 position is described below. This model showed very good predictive performances (R^2^ = 0.83).

$$\begin{aligned} Relative\, QS \,in\, H45-&K40\, (N/kg) = 8.13 + 0.35 \times relative \,QS \,in \,H90-K90 \,[N/kg] - 0.021 \times age \, [years] - 0.16 \times BMI \, [kg/m2] + \alpha \, \\ With \, \alpha & = 0.85 \times raw \, QS \, in \, H45-K40 \, [N] \,if \,raw \,QS \,in \,H45-K40 \,between \,362 \,and \,483N \\ & = 2.21 \times raw \,QS \,in \,H45-K40 \,[N] \,if \,raw \,QS \,in \,H45-K40 > 483N \end{aligned}$$

Relative QS in H90–K90 was significantly associated with relative QS in H45–K40 (p < 0.001) only. The other following parameters were not included in the model, as they were not associated with the dependant variable: age (p = 0.63), sex (p = 0.33), BMI (p = 0.26), raw QS in H45–K40 in moderate and strong tertiles (p = 0.45 and p = 0.36, respectively). The final equation estimating relative QS in H90–K90 position is described below. This model showed good predictive performances (R^2^ = 0.56).

$$Relative \,QS \,in \,H90-K90 \,(N/kg)\hspace{0.17em}=\hspace{0.17em}1.09\hspace{0.17em}+\hspace{0.17em}0.57\hspace{0.17em}\times \hspace{0.17em}relative \,QS \,in \,H45-K40 \,[N/kg]$$

**Fig. 2:**
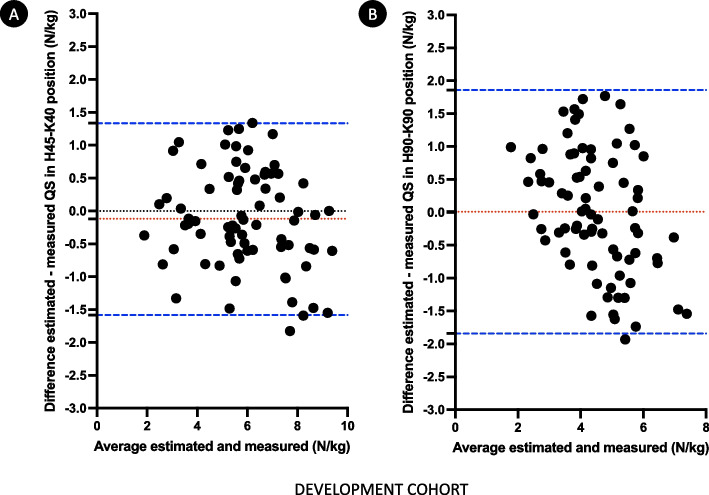
Bland–Altman plots showing the difference between estimated and measured relative QS in H45–K40 position (**A**) (bias − 0.12 N/kg, limits of agreement of − 1.58 N/kg and 1.34 N/kg) and in H90–K90 position (**B**) (bias 0.009 N/kg, limits of agreement of − 1.84 N/kg and 1.86 N/kg) in the development cohort. Mean differences are represented by red dotted lines, limits of agreement are represented by blue dashed lines

The QS in both positions were re-estimated according to the corresponding new equation. Results of the comparison between estimated and measured QS, and reliability assessment are presented in Table 2. No significant difference was observed between estimated and measured QS in both positions. The reliability was considered acceptable to excellent. Performances of equations compared to measurement are represented in Bland–Altman plots (Fig. 2).

**Table 2:** Comparison and reliability between estimated and measured relative QS in the development cohort (n = 77)

	Measured relative QS (N/kg)	Estimated relative QS (N/kg)	t-test p-value	ICC (95% CI)	Bias (limits of agreement)
H45–K40	5.9 [4.9–7.4]	5.8 [5.0–7.1]	0.15	0.91 (0.87–0.95)	− 0.12 (− 1.58–1.34)
H90–K90	4.4 [3.5–5.6]	4.5 [3.9–5.2]	0.94	0.73 (0.60–0.82)	0.01 (− 1.84–1.86)

ICC: interclass correlation coefficient; QS: quadriceps strength

Bias: mean of the differences between estimated and measured QS

As the order of the test was randomly assigned, we divided this cohort according to the first position used to measure QS (H45–K40 first followed by H90–K90, and vice versa), and we defined specific regression models in both subgroups, aiming to predict strength in the two positions. The generated models showed some minimal differences between subgroups in terms of coefficients, but their performances were similar: the R^2^ of the models estimating QS in H45–K40 was 0.84 in both subgroups, and the R^2^ of the models estimating QS in H90–K90 were 0.59 and 0.47 when the first tested position was respectively the supine and seated position. These equations were then used to re-estimate QS in both positions in the global cohort according to the defined subgroups: the reliability between estimated and measured relative QS were very similar to results detailed in Table 2. The ICC (IC95%) were 0.93 (0.89–0.95) and 0.73 (0.61–0.82) for QS in respectively H45–K40 and H90–K90 positions. Considering the order of the test for the further steps was thus irrelevant.

### Second part of the study: external validation of the new equations in healthy volunteers

In the validation cohort, 62 participants (33 males, 53.2%) were included. The median age of the subjects was 36.5 [27–53] years; median height, 170 [165–180] cm; median weight, 73 [65–83.5] kg; and median body mass index, 24.9 [22.8–27.4] kg/m^2^. No statistical differences were observed in terms of demographic data between the validation cohort and the development cohort. The measured QS in the two positions in the validation cohort are shown in Table 1. QS in H45–K40 and in H90–K90 were not statistically different (p = 0.323 for raw QS and p = 0.262 for relative QS). Raw and relative QS in H90–K90 position were significantly higher in this cohort compared to the development cohort (p < 0.001 and p < 0.001, respectively).

**Fig. 3:**
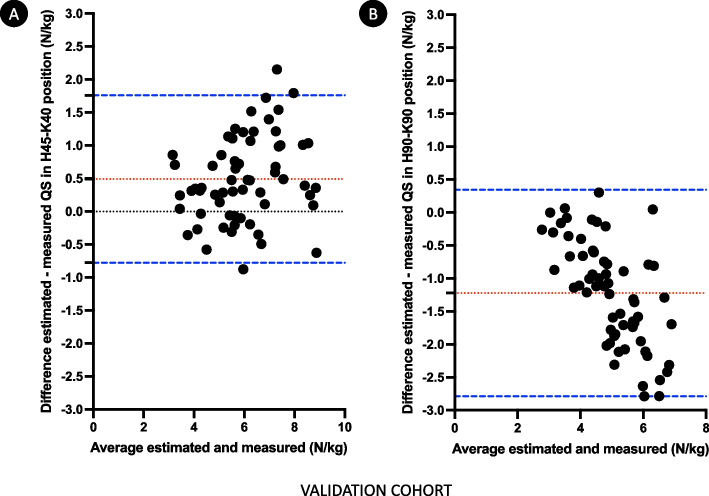
Bland–Altman plots showing the difference between estimated and measured relative QS in H45–K40 position (**A**) (bias 0.49 N/kg, limits of agreement of − 0.78 N/kg and 1.76 N/kg) and in H90–K90 position (**B**) (bias − 1.22 N/kg, limits of agreement of − 2.79 N/kg and 0.34 N/kg) in the validation cohort of healthy volunteers. Mean differences are represented by red dotted lines, limits of agreement are represented by blue dashed lines

The relative QS in both positions were re-estimated according to the corresponding new equation. Results of the comparison between estimated and measured relative QS, and reliability assessment are presented in Table 3. Statistically significant differences were observed between estimated and measured relative QS in both positions. Reliability and bias were considered acceptable only for the equation estimating the relative QS in H45–K40. The other equation, estimating relative QS in H90–K90 position, did not performed well in terms of ICC and bias. Performances of equations compared to measurement are represented in Bland–Altman plots (Fig. 3).

**Table 3:** Comparison and reliability between estimated and measured relative QS in the validation cohort of healthy volunteers (n = 62)

	Measured relative QS (N/kg)	Estimated relative QS (N/kg)	t-test p-value	ICC (95% CI)	Bias (limits of agreement)
H45–K40	5.7 [4.8–6.7]	6.1 [5.1–7.7]	< 0.001	0.87 (0.59–0.94)	0.49 (− 0.78–1.76)
H90–K90	5.8 [4.6 –6.5]	4.3 [3.8–4.9]	< 0.001	0.46 (− 0.10–0.77)	− 1.22 (− 2.79–0.34)

ICC: interclass correlation coefficient; QS: quadriceps strength

Bias: mean of the differences between estimated and measured QS

### Third part of the study: external validation of the new equations in ICU survivors

In this validation cohort, 50 participants (35 males, 70%) were included. Median age of the subjects was 70 [63–75] years; median height, 171 [165–177] cm; median weight, 80 [68.2–90.3] kg; and median body mass index, 27.2 [23.4–29.7] kg/m^2^. There was a statistical difference in terms of age, sex and BMI between the ICU survivors and the two cohorts of volunteers (respectively p < 0.001, p = 0.017, p = 0.006). Patients were tested 3 [2–5] days after ICU discharge, following an ICU stay of 2 [2–4] days. The measured QS in the two positions in this third cohort are shown in Table 1. QS in H45–K40 and in H90–K90 were not statistically different (p = 0.336 for raw QS and p = 0.422 for relative QS). Raw and relative QS in both positions were significantly lower in this cohort compared to the validation cohort of healthy volunteers (p < 0.001 for each of the four comparisons).

As the equation estimating the relative QS in H90–K90 position did not perform well in the previous validation cohort, only the relative QS in H45–K40 position was re-estimated according to the corresponding equation. Results of the comparison between estimated and measured relative QS, and reliability assessment are presented in Table 4. Statistically significant differences were observed between estimated and measured QS. The tested equation estimating relative QS in H45–K40 position did not perform well in terms of ICC and bias.

**Table 4:** Comparison and reliability between estimated and measured relative QS in the validation cohort of ICU survivors (n = 50)

	Measured relative QS (N/kg)	Estimated relative QS (N/kg)	t-test p-value	ICC (95% CI)	Bias (limits of agreement)
H45–K40	2.9 [2.3–3.6]	3.5 [3–4]	< 0.001	0.60 (0.24–0.78)	0.53 (− 1.01–2.07)

ICC: interclass correlation coefficient; QS: quadriceps strength; Bias: mean of the differences between estimated and measured QS

## Discussion

This study represents a first attempt to develop equations estimating QS in the supine position (H45–K40) based on a QS measurement in the seated position (H90–K90), and vice versa. We used a robust statistical method and three different cohorts of healthy volunteers and ICU survivors. Interestingly, when applied on frail patients, none of these equations performed well enough to be used in clinical or research practices.

To develop these new equations, we included in a multivariate regression model the main clinical parameters that are known to influence the generated quadriceps strength during isometric contraction (i.e., age, sex and BMI) [16, 17]. However, muscle strength is the consequence of a complex interaction between all neuromuscular elements, including neural, muscular and mechanical factors. These factors are not easily measurable in clinical practice, and thus could not be included in the multivariate model. The complexity of the muscle contraction and generated strength can explain the difficulties to develop high-performance equations for muscle strength estimation.

A huge heterogeneity in quadriceps strength has been observed among ICU survivors: some patients are extremely weak, whereas others are stronger than healthy patients [9, 18]. This observation reinforces the concept of “one size does not fit all” and assumes a need for individualized rehabilitation. The prerequisite is to measure muscle performances as early as possible to determine patients who will benefit from a tailored rehabilitation program. The first baseline measurement of QS strength is usually per-formed at bedside, in the supine position, due to constraints related to the ICU setting, patient’s medical condition and muscle weakness. During follow-up after ICU discharge, most often in an outpatient setting, QS is more easily measured in the seated position. As previously demonstrated, the two measures cannot be compared, and we have demonstrated that the two measurements cannot be converted (i.e., the conversion formula did not performed well enough in the validation cohort including patients). This has methodological implications for clinical and research purposes in case of repeated measurements: the tested position should be standardized and should be the same at all timepoints. Moreover, these considerations should be clearly mentioned in any clinical report or method statement.

One aim of the present study was to specify the modalities of functional assessment in ICU survivors, in particular the quadriceps strength measurement. This parameter is probably a key element for clinicians to evaluate physical improvements during post-ICU rehabilitation and follow-up. First, muscle strength measurement is a key component of the diagnostic and follow-up testing to, respectively, identify patients with ICU acquired weakness as early as possible and assess progresses during rehabilitation. Isometric dynamometry, unlike isokinetic testing (even if considered as the gold standard for strength measurement), seems technically and practically appropriate for these purposes. Muscle strength measurement is not the only method to assess muscle impairments during and after an ICU stay. Strength assessment is a complement to muscle mass measurement and functional assessment, as we know that mass, strength and function may not be perfectly correlated [19]. Second, quadriceps strength may be a better parameter for mobility and autonomy outcomes. If handgrip dynamometry is popular, cheap and easy to perform, handgrip strength may not be representative of global muscle strength in critically ill patients and survivors [20]. Third, quadriceps dynamometry, compared to manual muscle testing such as the Medical Research Council (MRC) scale, may have better sensitivity to strength change during longitudinal clinical assessment or studies [21]. QS measurement is not part of current guidelines or core outcome sets per se, but is increasingly evoked during discussions, such as during the “*CONCISE*” core outcome set development [22]. The ideal place of QS measurement as muscle health assessment in ICU survivors remains to be defined. However, this objective measure is particularly suited for longitudinal studies. In this case, once again, QS measurement should follow strict rules in terms of method, especially regarding body and leg positions, to provide accurate, reliable, and comparable values.

In this study, a limitation should be acknowledged: QS measurement in ICU survivors in the seated position were performed at the edge of the bed. This sitting surface were softer than the surface used in the volunteers’ cohorts. It is unlikely that this difference had impacted the generated strength, as hip and knee flexion angles were kept the same.

In conclusion, in this 3-step study, no equations allowing the estimation of relative QS in supine or seated position, based on a measurement in the other position, performed well enough to be used in post-ICU survivors. The present results help clarify the terms of use of quadriceps dynamometry in critically ill patients and during the post-ICU trajectory: QS should be strictly tested in the same position in case of repeated measurements. In the ICU, the conditions make it easier to measure QS in supine position for an early assessment, and further measurements should be performed in the very same position, even if patients could then be tested in other positions.

## Data Availability

The datasets used and/or analyzed during the current study are available from the corresponding author on reasonable request.

## Abbreviations

BMI: Body mass index
ICU: Intensive care unit
ICU-AW: ICU-acquired weakness
QS: Quadriceps strength
